# Assessment of Systemic Condition and Smoking Impact Over Incidence of Apical Periodontitis

**DOI:** 10.7759/cureus.55250

**Published:** 2024-02-29

**Authors:** Sorina G Zahiu, Ovidiu Fratila

**Affiliations:** 1 Doctoral School of Biomedical Sciences, University of Oradea, Oradea, ROU; 2 Department of Medical Disciplines, Faculty of Medicine and Pharmacy, University of Oradea, Oradea, ROU

**Keywords:** systemic condition, general health, endodontic infections, risk factors, apical periodontitis

## Abstract

This study aimed to assess the prevalence of apical periodontitis in a subset of the population of south-eastern Romania and to analyze the overall health status of the group of patients with apical periodontitis. The medical and dental history, including age, gender, background, presence of smoking, level of education, the total number of teeth present and with apical periodontitis, and the general health status were recorded from a total of 200 patients. The periapical status was analyzed using an orthopantomogram and periapical radiographs of teeth that were diagnosed with periapical lesions by the same dental professional. The periapical status was classified according to the periapical index (PAI), with apical periodontitis being present if the PAI score ≥3. The majority of patients were female (58.5%), with secondary or higher education from urban areas and the mean number of teeth with apical periodontitis was 2.29 ± 1.26, with a median of 2 teeth. A total of 17.1% of patients were smokers, these patients had two more teeth with periapical pathology, and 16% of all patients had general diseases, the most common of which was cardiovascular disease (8.2%). Compared with those without the disease, these patients had a higher number of teeth with apical periodontitis (median = 2.5, IQR = 2-4 vs. median = 2, IQR = 1-3). As a result, this scientific research suggests an association between smoking, cardiovascular disease, and gastritis with apical periodontitis, but no association could be demonstrated between apical periodontitis and other systemic diseases.

## Introduction

Apical periodontitis is considered a major health problem affecting the adult population in many countries [1]. Several epidemiologic studies have highlighted the prevalence of apical periodontitis, and have reported different values in countries like Spain (61.1% ), Kosovo (46.3%), Canada (44% and 51%), and Japan (69.8%) [2-5]. Apical periodontitis has had a huge impact over the last decade in both endodontically treated and non-endodontically treated teeth [6,7].

Apical periodontitis is a complex multifactorial disease that is caused either by infected necrotic pulp or by a failed endodontic treatment [7]. Failure to diagnose apical periodontitis on time may result in local and systemic complications [8]. Complications of apical periodontitis can be manifold, with reports of severe acute suppurations that are life-threatening infections of the maxillary sinus or the appearance of chronic inflammatory cysts that may invade adjacent anatomical structures [9,10].

Bacterial infection is the determining factor of apical periodontitis but in the literature, we have identified other contributing factors [11,12]. Older age is associated with a poor immune response. The prevalence of apical periodontitis increases with age: by the age of 50, one in two individuals will have the disease and in individuals over 60 years of age, the prevalence will increase to 62% [13].

Patient education and socioeconomic factors seem to have a significant impact on the incidence of apical periodontitis, but unfortunately, there are very few authors who have analyzed these issues [14,15].

General conditions may have a different interference with periapical lesions. There are diseases that modify the immune response (allergies, autoimmune diseases, liver diseases, blood diseases). Other diseases can change the metabolism (endocrine, hepatic, renal), having an impact on the healing process. Diabetes is well known for its vasculopathy, reduced tendency to heal, and tendency to exacerbate infections. There are also diseases that directly target metabolism and bone structure, such as osteoporosis, Paget's disease, etc. Systemic conditions and disorders may be seen as factors that modulate the progression of oral infections rather than serving as the primary cause [16].

Numerous studies have shown a link between endodontic infections and cardiovascular disease. Patients with apical periodontitis were found to be 5.3 times more likely to have cardiovascular disease than those without, and patients with poor glycemic control will have a higher number of periapical lesions compared to those with tight glucose control [17]. A limitation of these studies is derived from the fact that they often assess associations only between systemic disease and apical periodontitis, without considering other associated factors.

Nicotine is associated with many diseases and has been shown to have undesirable effects on the immune system. Smoking can have as a side effect the periodontium and the periapical area damage [18]. There are a few studies that have found an increased prevalence among smokers and others that have found no association between the two [19-22].

In a systematic review and meta-analysis, the association between apical periodontitis and inflammatory bowel disease was found positive [23]. Oral manifestations of inflammatory bowel disease can be found in 8%-10% of patients and can include decreased saliva production, caries, recurrent oral abscesses, gingivitis, angular cheilitis, and periodontal disease [24].

Studies on periapical health have not yet been performed in Romania. No data are available on the prevalence of apical periodontitis or general health status in these patients. The purpose of this study was therefore to determine the main characteristics of patients with apical periodontitis, allowing the identification of common risk factors for the development of this condition. Specifically, we wanted to find out if there is a link between age groups and the occurrence of periapical lesions. We were also interested in whether smoking statistically influenced the occurrence of apical periodontitis in the studied population group. The general condition of the patients was also a monitored parameter, we wanted to see the association of this diversified parameter with the periapical lesions. The link between the level of education and the occurrence of periapical conditions is also a parameter that it deserves to be analyzed.

## Materials and methods

Institutional review board statement

This study was performed in line with the principles of the Declaration of Helsinki. Approval was granted by the Ethics Committee of the Medical Faculty of the University of Oradea (No. CEFMF/4, 30.10.2023).

Sample selection

A total of 1,004 patients presenting for endodontic treatments at the study host clinic between January 3, 2020 and September 30, 2021 were available for the present study. Each subject completed an informed consent form and a detailed anamnesis form. After clinical and radiographic examination, a number of 200 patients required endodontic treatments for at least one tooth with apical periodontitis.

Inclusion criteria

All patients over 15 years of age who requested and received dental treatment were included in the study if they had at least one tooth with apical periodontitis confirmed on X-ray. Those with general pathologic conditions had to write the precise diagnosis given by the specialist doctor.

Exclusion criteria

Patients who had general pathologic conditions but did not have a clear diagnosis during the study were excluded. Also, patients who had a very small number of teeth (up to seven) in the oral cavity were not included in the study. These patients represented an unfavorable extreme in the relationship between ailments and care, similar to the terminal state. The wisdom teeth were not considered in our study because their pathology is more different than that of the rest of the teeth (pericoronaritis, inclusions). Pregnant women were excluded from the study due to their unjustified reluctance to take X-rays during pregnancy, as their files may be incomplete. Also, teeth with marginal periodontal damage and pockets of more than 4 mm or vertical bone loss of more than 5 mm were not taken into account due to the risk of pulpal damage secondary to marginal periodontal disease. Teeth with large or irreparable coronal destructions were also not included in the study.

Radiographic examination

In order to assess the number of teeth with apical periodontitis, they were clinically and radiologically examined using panoramic digital radiography. This is a screening method necessary for each dental patient but could have dimensional distortions [25]. A total of 1,004 panoramic radiographs (20,072 teeth) were analyzed at the initial imaging examination. All digital orthopantomographs were taken using the Kavo Orthopantomograph™ OP 3D digital device, made by the same dental professional according to the exposure parameters appropriate for the patient's gender, age, and weight. Subsequently, based on this information, in order to obtain a detailed evaluation of the periapical status, the teeth that were diagnosed with apical periodontitis were re-evaluated with periapical radiographs, using the parallelization technique (Carestream CS 2100 Intraoral X-ray and Sopro Imaging software). Proper protection equipment for each patient was used. Even if one patient needed four periapicals, the total irradiation dose including also the panoramic one was acceptable. A simple estimation of the absorbed dose for the intraorals was 4x 2.5µSv=10 µSv. The panoramic dose was less than 9 µSv. It means the total absorbed dose was less than 20 µSv [26]. This is the natural background dose for approx. 3 days considering the annual natural background dose is 2.4 mSv. All periapical radiographs were taken and interpreted by the same dental professional, in order to ensure homogeneity of results. The method of viewing the radiographs was standardized: the radiographs were examined using a computer, in a darkened room, where the ambient light was controlled for optimal contrast. The periapical status of all teeth was examined using the periapical index scoring system (PAI), proposed by Ørstavik et al. which scores the apical area of the radiographic images as follows: 1. normal periapical structures; 2. small changes in the bone structure; 3. changes in bone structure with some mineral loss; 4. periodontitis with a well-defined radiolucent area; 5. severe periodontitis with exacerbating features. A score of PAI 1 and 2 was defined as a normal periapical region and PAI scores (3, 4, and 5) were evaluated as apical periodontitis. One observer with 6 years of clinical experience in endodontics examined the radiographs. All radiographs were evaluated again to determine intra-examiner agreement with regard to the detection of periapical radiolucency and the Kappa coefficient was applied. In case of multi-rooted teeth, the biggest root with the biggest PAI score was chosen.

Clinical examination

Each patient completed the medical questionnaire and underwent a panoramic radiograph, followed by clinical examination. The clinical examination included an interrogation, an inspection, a palpation, a cold test, oral hygiene, percussion, and a periodontal probing. After confirming the diagnosis of apical periodontitis, through the clinical examination and the radiological evidence of the lesion, a periapical radiograph of the causal tooth was performed.

Measurable variables

Several categories of variables were followed for each patient as follows. General variables: background, gender, age in years, level of education (primary, secondary, high school, university), smoker or non-smoker. Variables related to the general diseases: cardiovascular, respiratory, gastrointestinal (gastritis), hepatic, neuropsychiatric, blood, immune, allergic, endocrine disorders. Variables related to dental status: number of teeth present on the arch, number of teeth with apical periodontitis.

Statistical analysis

Statistical analysis was performed by using SPSS Statistics 25 (IBM) and Microsoft Office Excel/Word 2021. Quantitative variables were tested for distribution using the Shapiro-Wilk test and were expressed as means with standard deviations or medians with interpercentile ranges. Independent quantitative variables with non-parametric distribution were tested using the Mann-Whitney U/Kruskal-Wallis H test, and any correlation between them should be checked using Spearman's rho correlation coefficient. The qualitative variables were expressed as absolute or as a percentage. Differences between independent qualitative variables were tested using Fisher's Exact/Pearson Chi-Square tests. Pair quantitative variables with non- parametric distribution were tested using the Wilcoxon test and paired qualitative variables were tested using the Marginal Homogeneity Test. The Dunn-Bonferroni post-hoc tests were used to refine the results obtained in testing independent quantitative variables. The Bonferroni-corrected Z-tests were used to refine the results obtained in the contingency tables. The McNemar post-hoc tests were used to refine the results obtained in testing paired qualitative variables.

## Results

A number of 200 patients met the conditions for inclusion in the study. They presented a total of 4,270 teeth in the dental arches, of which 385 teeth were identified as having apical periodontitis. The most common apical periodontitis was found in female patients with secondary or higher education from urban areas, the characteristics of the studied group are shown in Table 1.

**Table 1:** Characteristics of the studied patients No.: number of patients (200). The data is represented as number of cases with percentages for categorical variables and means with standard deviations along with medians with interquartile ranges for quantitative variables.

Parameter	Values (No., %)
Gender (No. = 200, %)	117 (58.5%) Female
	83 (41.5%) Male
Age (Mean ± SD, Median (IQR))	36.57 ± 14.47, 35 (25-47)
Environment of origin (No. = 200, %)	74 (37%) Rural
	126 (63%) Urban
Smokers (No. = 200, %)	34 (17%) Present
Level of education (No. = 200, %)	1 (0.5%) Primary
	5 (2.5%) Secondary
	92 (46%) High School/ Vocational School
	102 (51%) University education
Total number of teeth present (Mean ± SD, Median (IQR))	23.73 ± 4.82, 26 (21-28)
Total number of teeth with apical periodontitis (Mean ± SD, Median (IQR))	1.93 ± 1.17, 2 (1-2)
General conditions (No. = 200, %)	32 (16%) Present
Cardiovascular disease	16 (8%) Present
Respiratory conditions	2 (1%) Present
Gastrointestinal conditions	4 (2%) Present
Liver disorders	3 (1.5%) Present
Neuropsychiatric disorders	5 (2.5%) Present
Blood disorders	2 (1%) Present
Immune disorders	3 (1.5%) Present
Allergic diseases	6 (3%) Present
Endocrine disorders	4 (2%) Present
No. of diseases (No. = 200, %)	169 (84.5%) Absent
	21 (10.5%) One condition
	8 (4%) Two conditions
	2 (1%) Three conditions

**Figure 1:**
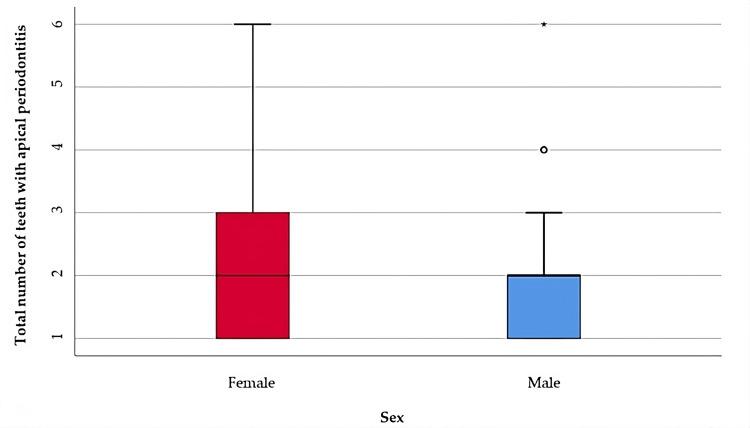
Comparison of the total number of teeth with apical periodontitis in relation to gender The data is represented as medians with interquartile ranges in the box-plot figure. For the illustration of the quantitative values distributions in the box-plot graphs, the SPSS Statistics software illustrates any values that are below the 1st quartile (25th percentile) – 1.5* interquartile range or above the 3rd quartile (75th percentile) + 1.5*interquartile range, as outliers represented by circles in the graph. As for values that are below the 1st quartile (25th percentile) – 3* interquartile range or above the 3rd quartile (75th percentile) + 3*interquartile range, the software represents the values as extreme outliers represented by asterisk symbols in the graph.

Table 2 and Figure 1 include the data of the analysis of the distributions of apical periodontitis cases according to the number of teeth affected and the patient gender.

**Table 2:** Comparison of the total number of teeth with apical periodontitis in relation to gender *Mann-Whitney U Test **Shapiro-Wilk Test, the data is represented as means with standard deviations along with medians with interquartile ranges, p-value considered significant at values below 0.05 (p<0.05).

Gender	Mean ± SD	Median (IQR)	Mean Rank	p-value*
Female (p<0.001**)	1.97 ± 1.24	2 (1-3)	101.56	0.739
Male (p<0.001**)	1.86 ± 1.07	2 (1-2)	99.00	

The distribution of the total number of teeth was non-parametric in most groups according to the Shapiro-Wilk test (p<0.05). The differences in the total number of teeth with apical periodontitis observed in the gender-distributed groups were not statistically significant according to the Mann-Whitney U test (p= 0.739), so the gender of the patients did not significantly influence the total number of teeth with apical periodontitis.

**Figure 2:**
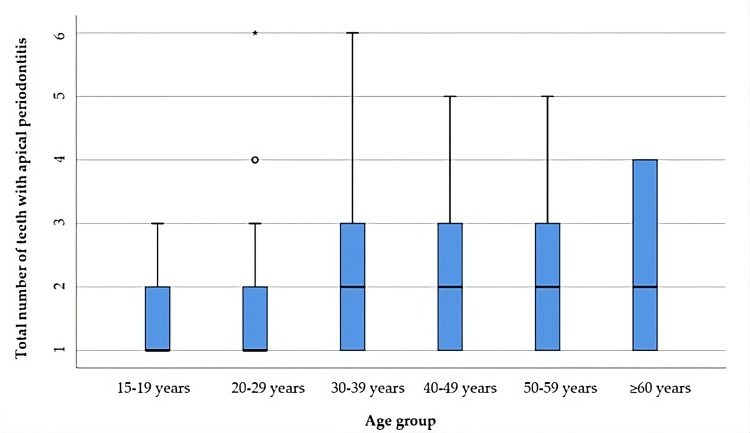
Comparison of the total number of teeth with apical periodontitis by age group The data is represented as medians with interquartile ranges in the box-plot figure.

The maximum incidence of periodontitis cases is in middle-aged adults, it can be seen from Tables 3-4, and Figure 2 represents the comparison of the total number of teeth with apical periodontitis in relation to age categories. The distribution of the total number of teeth was non-parametric in most groups according to the Shapiro-Wilk test (p<0.05). The differences in the total number of teeth observed in the age-distributed groups are statistically significant according to the Kruskal-Wallis H test (p=0.005), and the post-hoc tests only show that patients aged 15-19 years had a significantly lower number of teeth with apical periodontitis compared to patients aged 50-59 years (p=0.024).

**Table 3:** Comparison of the total number of teeth with apical periodontitis by age group *Kruskal-Wallis H Test **Shapiro-Wilk Test, the data is represented as means with standard deviations along with medians with interquartile ranges, p-value considered significant at values below 0.05 (p<0.05).

Age Group	Mean ± SD	Median (IQR)	Mean Rank	p-value*
15-19 years (p<0.001**)	1.32 ± 0.55	1 (1-2)	72.64	0.005
20-29 years (p<0.001**)	1.68 ± 1.1	1 (1-2)	87.54	
30-39 years (p<0.001**)	2.22 ± 1.41	2 (1-3)	110.62	
40-49 years (p<0.001**)	2 ± 0.98	2 (1-3)	109.96	
50-59 years (p=0.001**)	2.31 ± 1.28	2 (1-3)	118.86	
≥60 years (p=0.003**)	2.21 ± 1.31	2 (1-4)	112.54	

**Table 4:** Post-hoc comparison of the total number of teeth with apical periodontitis by age group *Dunn–Bonferroni Post-Hoc Test, p-value considered significant at values below 0.05 because of their written adjusted values.

Age*	15-19 years	20-29 years	30-39 years	40-49 years	50-59 years	≥60 years
15-19 years	-	1.000	0.094	0.103	0.024	0.388
20-29 years	1.000	-	0.623	0.690	0.157	1.000
30-39 years	0.094	0.623	-	1.000	1.000	1.000
40-49 years	0.103	0.690	1.000	-	1.000	1.000
50-59 years	0.024	0.157	1.000	1.000	-	1.000
≥60 years	0.388	1.000	1.000	1.000	1.000	-

**Figure 3:**
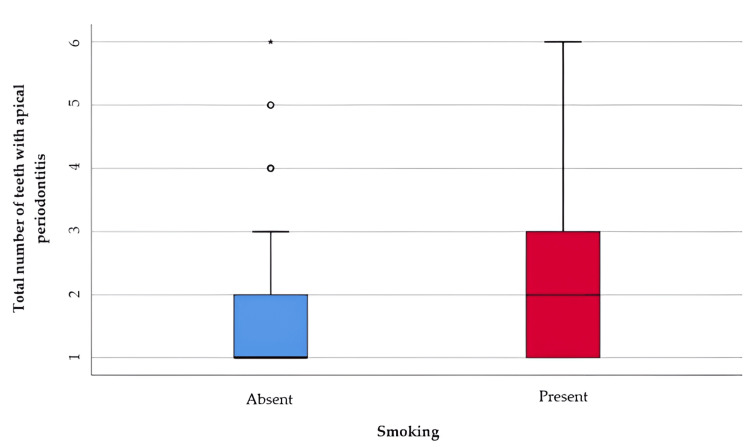
Comparison of the total number of teeth with apical periodontitis of patients according to smoking The data is represented as medians with interquartile ranges in the box-plot figure.

The influence of smoking on the number of teeth present on the arch and the incidence of apical periodontitis is expressed in the data in Table 5 and Figure 3 represents the comparison of the total number of teeth with apical periodontitis of patients according to smoking status. The distribution of the total number of teeth with apical periodontitis is non-parametric in both groups according to the Shapiro-Wilk test (p<0.001). According to the Mann-Whitney U test, smoking patients had a significantly higher number of teeth with apical periodontitis (median = 2 teeth, IQR = 1 - 3 teeth) compared to non-smoking patients (median = 1 tooth, IQR = 1-2 teeth) (p=0.031).

**Table 5:** Comparison of the total number of teeth with apical periodontitis of patients according to smoking status *Mann-Whitney U Test **Shapiro-Wilk Test, the data is represented as means with standard deviations along with medians with interquartile ranges, p-value considered significant at values below 0.05 (p<0.05).

Smoking	Mean ± SD	Median (IQR)	Mean rank	p-value*
Absent (p<0.001**)	1.85 ± 1.14	1 (1-2)	96.31	0.031
Present (p<0.001**)	2.29 ± 1.26	2 (1-3)	117.93	

**Figure 4:**
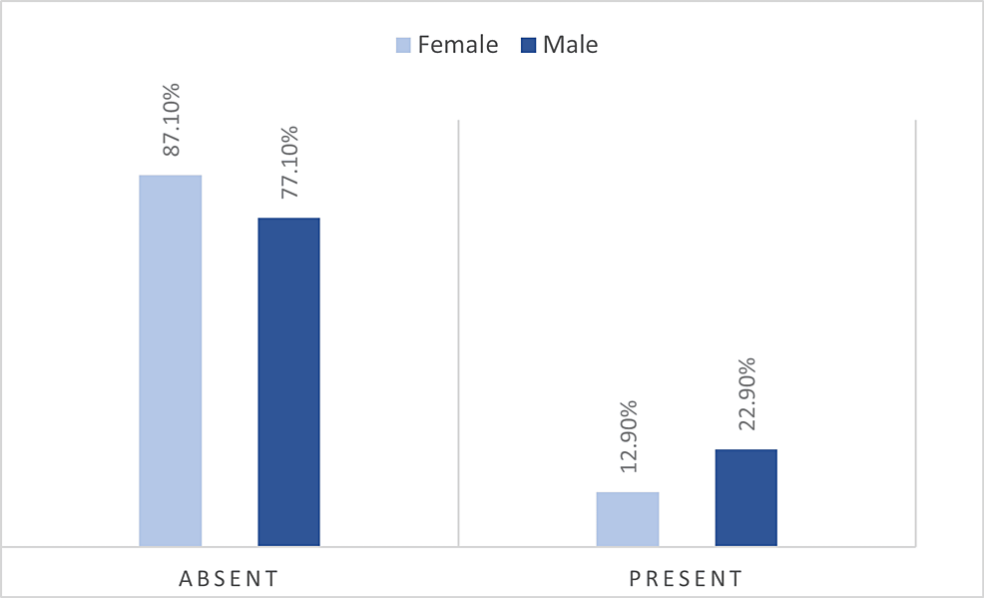
Distribution of patients by gender and smoking status The data is represented as number of cases with percentages.

The data in Table 6 and Figure 4 represent the distribution of patients by gender and smoking status. Differences in smoking frequency between genders are not statistically significant according to the Pearson Chi-Square test (p=0.066), however they show a trend toward statistical significance in the sense of a possible association between male gender and a higher smoking frequency.

**Table 6:** Distribution of patients by gender and smoking status *Pearson Chi-Square Test, the data is represented as number of cases with percentages, p-value considered significant at values below 0.05 (p<0.05).

Gender/Smoking	Female		Male		p-value*
	No.	%	No.	%	
Absent	101	87.1%	64	77.1%	0.066
Present	15	12.9%	19	22.9%	

**Figure 5:**
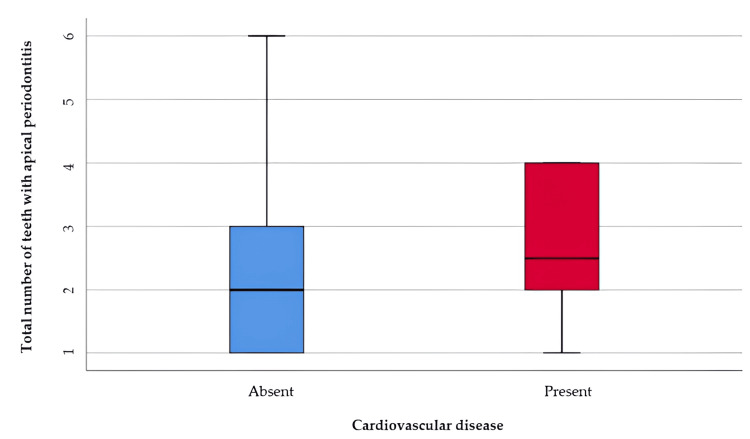
Comparison of the total number of teeth with apical periodontitis in relation to the presence of cardiovascular disease The data is represented as medians with interquartile ranges in the box-plot figure.

Among the patients included in the study, cardiovascular diseases showed the highest incidence, the analysis of cases with cardiovascular diseases and apical periodontitis is shown in Table 7 and Figure 5 represents the comparison of the total number of teeth with apical periodontitis reported to the existence of cardiovascular diseases. The distribution of the total number of teeth was non-parametric in both groups according to the Shapiro-Wilk test (p<0.05). The differences in the total number of teeth with apical periodontitis observed in the groups distributed according to the existence of cardiovascular diseases were statistically significant according to the Mann-Whitney U test (p=0.045), with a higher value of the number of teeth with apical periodontitis associated with cases with cardiovascular disease compared to those without diseases (median = 2.5, IQR = 2-4 vs. median = 2, IQR = 1-3).

**Table 7:** Comparison of the total number of teeth with apical periodontitis in relation to the presence of cardiovascular disease *Mann-Whitney U Test **Shapiro-Wilk Test, the data is represented as means with standard deviations along with medians with interquartile ranges, p-value considered significant at values below 0.05 (p<0.05).

Cardiovascular Disease	Mean ± SD	Median (IQR)	Mean Rank	p-value*
Absent (p<0.001**)	2.32 ± 1.42	2 (1-3)	136.01	0.045
Present (p<0.001**)	2.77 ± 1.24	2.5 (2-4)	167.87	

**Figure 6:**
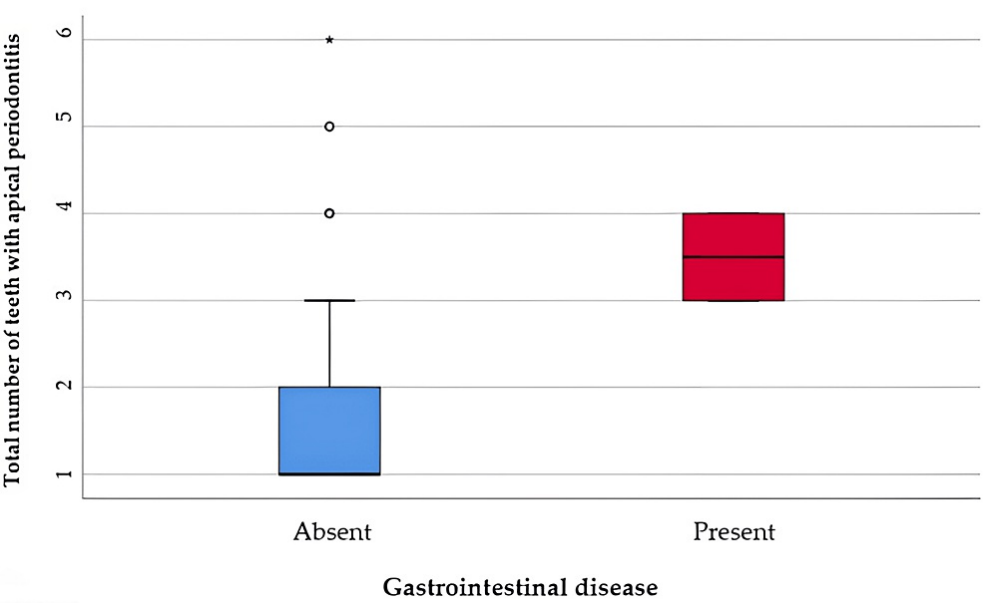
Comparison of the total number of teeth with apical periodontitis in relation to the presence of gastritis The data is represented as medians with interquartile ranges in the box-plot figure.

Analysis of cases with gastrointestinal disease - gastritis and apical periodontitis is shown in Table 8 and Figure 6. The distribution of the total number of teeth was non-parametric in both groups according to the Shapiro-Wilk test (p<0.05). According to the Mann-Whitney U test, patients with gastritis had a significantly higher number of teeth with apical periodontitis (median = 3.5 teeth, IQR = 3-4 teeth) compared to patients without gastritis (median = 1 tooth, IQR = 1-2 teeth) (p=0.006).

**Table 8:** Comparison of the total number of teeth with apical periodontitis in relation to the presence of gastritis *Mann-Whitney U Test **Shapiro-Wilk Test, the data is represented as means with standard deviations along with medians with interquartile ranges, p-value considered significant at values below 0.05 (p<0.05).

Gastrointestinal Disease	Mean ± SD	Median (IQR)	Mean Rank	p*
Absent (p<0.001**)	1.89 ± 1.16	1 (1-2)	99.01	0.006
Present (p=0.024**)	3.5 ± 0.57	3.5 (3-4)	173.50	

In the remaining cases, the systemic diseases did not significantly influence the presence of apical periodontitis.

## Discussion

This study considered patients over 15 years of age who presented themselves for endodontic treatments and in whom apical periodontitis was discovered. These were diagnosed by radiological screening and clinical examination and then confirmed by precision radiography. The patient's general conditions were taken into account for statistical association with the periapical lesions.

In our study, the patients most affected by the presence of apical periodontitis were female (58.5%), but this difference did not prove to be statistically relevant (p= 0.739), which was confirmed by other studies in the literature carried out in several countries in a similar geographical area. In contrast to our results, other authors identified a higher incidence of apical periodontitis in male patients [27,28]. This difference can be explained by the fact that female patients in our study showed a higher tendency to present for medical treatments (they are almost 2/3 of all patients).

The literature shows that a low level of education is a risk factor for the presence of apical periodontitis. In our study, the majority of patients have university studies. Similar results were reported by Aleksejuniene et al. who demonstrated that there is an association between an increased level of education and the presence of apical periodontitis [15]. It is quite possible that the result obtained by us is influenced to a large extent by socioeconomic factors. In the geographical area where the study was conducted, medical education and the concept of prophylactic treatment is still in its infancy, which means that even if patients with apical periodontitis have university education, it is very possible that they may not have any medical education. Another explanation could be given by the patients' attitudes toward treatment, most likely educated patients wanted to treat their teeth with apical periodontitis, unlike the less educated ones who ignored the condition and did not go to the doctor for a medical check-up.

The design of this study does not allow an evaluation of patients with apical periodontitis according to their ethnicity or genetics. However, in recent years this topic has begun to arouse interest in the scientific community. Thus, in a study published in 2023, Petty et al. identified a polygenic risk score that shows a certain degree of association with the occurrence of apical periodontitis, but without being able to associate a certain genetic phenotype in patients with apical periodontitis [29]. On the other hand, studies carried out in other regions of the globe reveal results similar to those obtained in the current study, thus in a study carried out in Brazil, Ferreira et al. identified a higher incidence of apical periodontitis in female patients [30]. In another study conducted in Sweden, Virtanen et al. identified a statistical association between apical periodontitis and cardiovascular disease [31]. Although from the studies analyzed by us up to this moment, we haven’t found that there is a causal association between a certain systemic condition and the appearance of apical periodontitis, it still seems that sexual dysmorphism influences the risk of occurrence of apical periodontitis [29].

The maximum incidence of apical periodontitis cases was found in middle-aged patients in contrast to other studies that incriminated older age as a predictor for apical periodontitis [14,27]. The results presented in this study also showed that the number of teeth with apical periodontitis is higher in older patients compared to younger patients. A possible explanation for this slightly different result from the literature may be that older patients with apical periodontitis prefer tooth extraction to conservative treatment or that their teeth can no longer benefit from conservative treatment. This is also a limitation of this study, which included patients who could benefit from the treatment of teeth with apical periodontitis.

The results of the study showed that smoking favors the occurrence of apical periodontitis (p= 0.031), which was confirmed by other studies in the scientific literature that showed an important association between its presence and apical periodontitis [32,33]. The impact of smoking appears to be significant in favor of the occurrence of apical periodontitis, with smokers having on average two more teeth with apical periodontitis than non-smokers, most likely because smoking also favors other oral diseases such as periodontal disease, tartar deposits that may indirectly favor the occurrence of apical periodontitis. In contrast, authors such as Bergström et al. reported that there would be no association between the two [22].

Cardiovascular disease refers to a range of conditions that affect the heart and blood vessels, including coronary artery disease, heart failure, and stroke [34]. Risk factors for cardiovascular disease include high blood pressure, high cholesterol, smoking, diabetes, obesity, and a sedentary lifestyle [35]. Chronic inflammation associated with apical periodontitis may contribute to the development or exacerbation of cardiovascular disease through mechanisms such as systemic inflammation and endothelial dysfunction [36]. It has been suggested that bacteria from dental infections, including apical periodontitis, could enter the bloodstream and potentially contribute to the formation of atherosclerotic plaques or trigger inflammatory processes within the blood vessels, thereby increasing the risk of cardiovascular events [37]. Numerous studies published in recent years have reported that there is an association between cardiovascular disease and apical periodontitis [38,39]. In our study, the analysis of the association between cardiovascular disease and apical periodontitis proved positive.

Inflammatory bowel disease is a group of inflammatory conditions of the colon and small intestine, Crohn’s disease and ulcerative colitis being the main phenotypes. Both conditions are chronic, multifactorial, of unknown origin, and marked by recurrent inflammatory processes affecting the gastrointestinal tract. Characterized by widespread inflammation, inflammatory bowel disease manifests through clinical episodes that mirror intestinal inflammation [40]. When the disease is active, patients tend to produce higher levels of pro-inflammatory molecules, which may contribute to the development of apical periodontitis [41,42]. Inflammatory bowel disease is associated with the occurrence of chronic active gastritis [43]. Few studies have found an association between apical periodontitis and gastrointestinal disease. Subjects with inflammatory bowel disease had a greater number of teeth with apical periodontitis. In our study, we found that patients with gastritis had a higher number of teeth with apical periodontitis (3-4 teeth). Other studies haven't provided data on the association between apical periodontitis and gastritis.

There are several limitations to this study: our assessment was based only on digital radiographs, which provide insufficient details about the presence of small periapical lesions that could be detected by using cone-beam computed tomography, and the fact that the study included a limited number of patients from a single geographic area.

## Conclusions

In this sample population, it was found that the highest incidence of apical periodontitis occurred in middle-aged patients. The lack of medical education and prophylactic treatments can lead to an increased prevalence of this pathology even in patients with higher education. The findings presented in this research suggest a link between the presence of smoking, cardiovascular disease, and gastritis with apical periodontitis. No correlation was established between apical periodontitis and other systemic diseases. To have more conclusive evidence regarding the association between general conditions and apical periodontitis, studies on larger groups of patients are required.
